# Clinical Examination of the Urine

**Published:** 1853-05

**Authors:** John Locke

**Affiliations:** The President, Professor of Chemistry and Pharmacy, in the Medical College of Ohio


*Clinical Examination of the Urine.* A Paper read before the Cincinnati Medical Society, Feb. 1st. 1853. By the President, JOHN LOCKE, M.D., Professor of Chemistry and Pharmacy, in the Medical College of Ohio. and Published by request of the Society.

*Gentlemen of the Cincinnati Medical Society.*

IN taking the seat with which your partiality has honored me, a seat to which I neither aspired, nor thought myself qualified to occupy, it would be inconsistent with the emotions I feel, not to make my acknowledgements for the distinction which you have, as I am informed, unanimously conferred upon me. I cannot but feel that it were more fitting that some practitioner, learned in authorities, and learned in the experience of years, and at the same time familiar with the minutiæ of the varying epidemics of daily practice, such men, indeed, as I see are by no means wanting in this body, were placed at the head of this society. Indeed, gentlemen, permit me to allude to what you all know and feel that we can scarcely expect to find, a better model than my very gentlemanly predecessor in office.*

Be assured that I hold the office at your daily pleasure, and will leave it quietly and willingly, whenever I know that there is a single dissenting emotion.

My devotion is to physical science, but I so strongly approve of the course of professional improvement which this society has pursued, that I shall abstain from any movement inclining to change its direction. *A society for mutual improvement in the knowledge and practice of the profession of Medicine*, seems to be the acknowledged motto, and while you confine yourselves to this, you cannot go wrong. The world is full of persecution aud violence, and it is hardly possible not to take the part of which we conceive to be the injured party. But those wrongs in the professional world, get righted by a little time and patience; and although as individuals we have the right of following the dictates of our judgment and feelings with regard to professional injuries, yet, as a society, it seems to me better in our official action to *avoid being used*, by even the injured. Let us remember that we are not a court of honor for the settling of professional difficulties, a species of Quixotism which would soon destroy itself, without rendering any service to human happiness. Almost every meeting which I have attended has been a model, and the quiet, yet able, manner in which medical subjects have been discussed has reflected honor upon our members, and has lighted up their countenance with an expression of happiness, growing out of conscious improvement. They have evidently felt themselves becoming wiser and happier.

*Dr. Richards.

How interesting it has been to see the profession come together, to confess their sins in cases in which they have failed of success, and to make common stock of the knowledge of the means by which they have been successful in relieving distress and lengthening life! How interesting it has been to see the young practitioner come forward, state a case and the difficulties, and ask for instruction, and how prompt is that instruction afforded by the veterans of our city. One is inclined to ask, is it possible that all this is done in the meridian of Cincinnati, that city in which the profession are supposed to be so hostile to each other? Permit me to congratulate the society, and say to you go on in your good works. Could the community see and know what you are doing without your parading it ostentatiously, it would tend to restore that confidence, which I much fear, has been lost, not without some just cause. By industry and true professional worth you will overthrow charlatanry without making the least demonstration of our attack upon its representatives.

Permit me to change my subject from that of Natural History, as proposed, to one more intimately connected with the practice of medicine. The diagnosis of diseases by the clinical examination of urine.

I present this subject, not because I am master of it, but for the two reasons, that it has been so much improved of late, that it presents itself almost as a new branch of knowledge, and because it is neglected by the profession, though not entirely; for one of our members, Dr. Buckner, alluded to some very properly conducted experiments, at our last, very interesting meeting. Our means of diagnosis of disease, are too limited at best, and even the means which actually exist, are often so partially used, that the most grave errors are committed, both with regard to the nature and the locality of disease. Our secretary mentioned to me this morning the case of a lady, who had been treated for pleurisy, when the passage of a large number of gall-stones showed the disease to have been hepatic. I myself knew a man to have been treated, through a lingering decline, for disease of the heart; when on the post mortem, it appeared that his heart and lungs were sound, but I took 96 large gall stones from the gall bladder, one being lodged in the duct, entirely obstructing it, the bladder itself being distended with bile, and the whole body being deeply tinged by the same substance. Cases of error in diagnosis are so numerous, that it is useless to begin to enumerate them. Every practitioner has read the tales which have been told by post-mortem revelations. Many, very many physicians make a very hasty and imperfect examination of their patients, doing little more than feeling the pulse and looking at the tongue, things which he does so mechanically, that he would not be unlikely to do them if called to a patient who had merely "got a flea in her ear," A mathematician applies his formulæ so skillfully that when he has reduced a problem he comes to an exact and perfect result. But what physician can see disease in its true and exact forms, through the dark and imperfect medium of diagnostic symptoms. The chemist can analyze a new compound, and can obtain his results with all of the exactitude of which the finest balance is susceptible, but who can rest satisfied with his diagnosis of an obscure case. With the doubting Christian, he must say, "Lord, I believe, help thou mine unbelief."

The fact is, that each case of disease is a problem of research in itself, and calls for a physician to be an acute philosopher, who can apply not only all known means of investigation, but that he shall be skillful in observing all new phenomena, and drawing just conclusions from them. The countenance, expression, color, temperature, dryness, moisture, touch, pulse, auscultation, percussion, local pains, color and appearance of feces ; and we may add, color and appearance of the urine, and we may also add, any change of the system from its normal condition, may be used for diagnosis. Many practitioners inspect the fecal discharges, who pay no attention to the urine, likely from a prejudice which they have taken against one of the lowest classes of impostors who pretend to vastly more than can possibly be ascertained from the urine, and at the same time, have no absolute skill to determine what may really be known from that secretion.

In order to be enabled to draw diagnostic conclusions from the condition of the urine, it needs a certain degree of knowledge of its chemical constituents, at least the proximate constituents, and the simpler modes of experimenting upon the subject.

The first points then, are to know what healthy urine is, and how to ascertain its deviations from what may be considered the latitude of its healthy standard.

In doing this, I must recur to elementary principles, and although the apparatus is of my own modelling, and the descriptions I shall give, are from nature, and direct from personal observation; yet I do not claim for this paper, anything more than an attempt at instructing our society in that which has been in general, already known. We need a work, in which all normal conditions should be pointed out, and at the same time the pathological condition correspondent to them, should be stated and the treatment indicated. No such work exists, except very partially, and I must rest satisfied with trying to make some points of this subject tangible to your senses.

The examination of urine may be divided into several distinct manipulations, as follows :

1. Inspection.
2. Acidity or alkalinity by tests.
3. Specific gravity.

4. Inspection after standing—Mucus, &c.
5. Heat—Albumen.
6. Evaporation.
7. Cooling—Urate of Ammonia.
8. Process for Urea.
9. Process for Uric Acid.
10. Examination for sugar.

1st. *Inspection.*—As early as possible after the urine is discharged, it should be inspected by being held up in a clear glass vial or beaker* between the eye and the light of a window. In health, it should be clear of any solid material and of a light amber color.

2d. *Acidity or Alkalinity by tests.*—The state of the urine as regards free acidity, can be determined by means of litimus paper. This paper may be prepared in the following manner.

Having purchased some solid litmus, 4 drachms to an ounce, dissolve it in distilled water, boiling if necessary, in a Florence flask. Place the blue liquid thus obtained, in a common saucer, and having prepared ribbons of white filtering paper, or any clean white bibulous paper, (printing paper will answer,) about three-fourths of an inch wide, draw them skilfully through the liquid and lay them on a frame of sticks or netting of twine stretched over a frame, in order to dry them. If the color be not deep enough, repeat the process of dipping and drying. Take a part of these blue test papers, thus prepared, and dip them in a solution of acid made as weak as possible, to change the blue to an evident red. A few drops of vinegar in a half pint of distilled water, may be tried, and the acidity increased if necessary, to accomplish the purpose. As acetic acid is volatile, a single drop of sulphuric acid in half a pint to a pint of water may be substituted. These reddened papers when dried, are to be preserved as tests for alkalinity, while the blue papers are to be used as tests for acidity. If, on dipping the end of the blue paper into the urine, it is reddened, the urine is determined to be *acid.* If no change is affected on the blue paper, then the reddened paper is to be inserted in the same manner, when, if the red color be changed back again to blue, the urine must be pronounced *alkaline.* If no change is effected on either of the papers, the urine is neither alkaline nor acid, under which circumstances, it is termed *neutral.*

The acidity of the urine, even in health, is continually varying, and its changes have a connexion with the condition of the stomach. Dr. Bence Jones says: "I found that the urine, passed the longest after food, is the most acid."

The general law to which the results obtained by Dr. Jones seem to point is,—that as acidity is entering into the stomach to

*A tumbler or similar glass.

perform the work of digestion, the acidity of the urine is dismissed, and sometimes, even alkalinity is produced; but digestion being performed, and the necessity of acid in the stomach ceasing, the acid is then carried off by the kidneys, and shows itself in the urine. *Almost always, the urine in health is positively acid*, and is merely reduced to a minimum of acidity during digestion, without reaching alkalinity.

*Caution.*—Practitioners who have got a very slight idea of urinary pathology, are apt to infer, that whenever the urine is acid, they must administer alkalies, and whenever it is alkaline, they must give acids. This erroneous idea might lead them to change their treatment of the same patient six times in one day, while he was at the same time in a state of continued and perfect health. I refer the reader to Dr. H. B. Jones's "Lectures on Animal Chemistry, in its application to stomach and renal diseases," for the method of determining the *degree* or *quantity* of acidity in the urine, in any given case; this being done on the general principle of measuring the quantity of alkali (carb. soda) found necessary to bring a given quantity of urine to a state of neutrality.

By means of the test papers, above described, the operator may determine whether the alkalinity of a specimen of urine, be due to fixed, or to the volatile alkali. When the volatile alkali is present, the blue color restored to the red paper, will disappear on drying and warming the paper so as to expel the ammonia which has changed it. This distinction is important in diagnosis and practice. If the urine be alkaline, by fixed alkali, mineral acids, and tonics have the best effect. But if carbonate of ammonia be found in the urine, *as it is discharged*, inflammation of the mucus membrane may be inferred.

3d. *The specific gravity* of urine, is an element of great importance in pathology; and as it is easily obtained, it should scarcely be omitted. The most ready way of obtaining specific gravity, is by an instrument made especially for the purpose, called the Urinometer. This instrument can be obtained of Mr. Foster, Optician and Phil. Inst. Maker, on Walnut street, between Third and Fourth streets, Cincinnati, at the price of about one dollar, in a wooden case suitable for the pocket. It is made of glass, and is only a modification of the common hydrometer. It is to be dropped into the urine after it is perfectly cold, (60° is the best temperature,) and the point on the scale of the stem, to which it sinks, indicates the specific gravity. The scale is graduated from zero. (0) indicating pure water, upward to 60, and is figured at every 10 degrees, as, 0, 10, 20, 30, &c. But these readings are understood to have 1000 prefixed to them as follows:—10 implies 1010; 20, 1020, &c. Twenty, viz: 1020, is about the mean standard of health; while 1030 to 1060, indicate the presence of sugar, and that diabetes melitus is the disease to be encountered, still there may be other solid materials in solution in the urine, which will increase the specific gravity to 1030, or upwards. Excess of urea may do this. The specific gravity of urine may be determ'ned by the use of the thousand grain bottle, but not with so much facility as with the urinometer.

*4th. Inspection after standing for several hours, say* 12 *to* 24. In healthy urine there will be a deposit of the mucus from the bladder, which is uslly semi-transparent, and appearing like an undefined haze in the lower part of the vessel. The quantity is variable, being increased by the slightest irritation of the bladder, scarcely amounting to disease. This secretion of mucus from the bladder, is in this particular, of variableness, not unlike the mucus secretion from the nose,

By far, the most usual deposit, after several hours, is that of the urate or lithate of ammonia. This appears as an amorphous sediment, varying in color, from nearly white, to a plain drab, reddish brown, or sometimes, rather a bright pink. It can be distinguished from most other deposits, by the facility with which it dissolves and disappears upon heating, or even warming the urine containing it, which may be accomplished domestically, by means of a spoon held over a candle, or better, in the laboratory, by a watch-glass, held over the spirit lamp, by the iron capsule hereafter described.

This deposit, urate of ammonia, often supervenes, upon slight disturbances of the stomach, and depends, in general, on a disproportion between the powers of the stomach, and the food supplied to it. The stomach may be in a healthy condition, but food and drink, excessive in quantity, or unsuitable in quality, may be forced upon it, overtaxing its powers, especially if little exercise be taken at the time, or the food and exercise may be suitable, and at the same time, the stomach may be to weak to complete the digestion. In either case, urate of ammonia will be likely to exist in excess, in the urine. Any condition which calls upon the kidneys to abstract from the system an excess of nitrogenized material, may develope urate of ammonia. Hence, a slight check of perspiration, by stopping the cutaneous outlet of nitrogonized matter, and throwing it upon the kidneys, is often followed by excessive deposites of urate of ammonia*. As urate of ammonia and urate of soda both contain, of course, uric or lithic acid, they are included by Dr. Bird, under the head of the uric acid diathesis. As urate of am monia is the most common of the urinary deposits, so it is that concerning which, most errors are committed by practitioners.

When it is light-colored, it is not unfrequently mistaken for pus, and especially when the urine is discharged, as sometimes happens, loaded, almost thick with this deposit. From pus it is

*See Dr. Golding Bird's Treatise on Urinary Deposits; section 139.

immediately distinguished by its solubility by heat. When of a red color, it is mistaken for the "lateritious sediment," "red sand," or uric acid. From this also, it is distinguished by its solubility by heat. Lithic acid crystals sometimes accompany urate of ammonia, and when this is the case, those crystals can be seen by dissolving the cloud of urate of ammonia by heat, or by allowing the red sand to settle to the bottom, which it naturally does, and decanting carefully the other deposites, proceed to wash the crystals with water; allowing them to settle at each washing, and decanting the fluid, carefully preserving the sediment. The term "brickdust sediment," gives a wrong idea of the crystals of uric acid, except as regards color. "Red sand," is a better term, for like sand, it is heavy, gritty and soluble; but unlike sand, the grains are mostly elongated, sharp pointed, spicular crystals, the form of which can be seen by the naked eye, or by a single lens of 2 inch focus.

A popular error in practice is, that whenever there is a deposite of urate of ammonia, the practitioner proceeds to administer alkaline remedies.

With regard to the use of alkaline remedies, the language of Dr. H. B. Jones is singularly clear and forcible*. I quote his remarks entire:

"The first test to be used is litmus paper. The question you ask, is—what is the state as regards acidity—not as regards the quality only, but as to the quantity? Is it too much, or too little acid? Litmus paper cannot fully answer this question. It can tell whether the urine is ammonical, or alkaline from fixed alkali, or contains little or much acid, but it cannot tell whether the acidity is more than it should be. Simple inspection of the urine, is able to solve this question and that better than any other mode, whatever. There cannot be an excess of free acid in the urine, without the uric acid being set free; though this often requires many hours to crystalise out. If then, you wish to know if the urine is too acid, you must leave the vial to rest for twenty-four, and sometimes ninety-six hours; and, if there be to much acid, red crystals of uric acid will be distinctly seen adhering to its sides, or deposited. The microscope may tell you quicker, but it will not tell you more surely than the naked eye. Whatever the degree of the reddening of the litmus, or the amount of the urate of ammonia sediment, you cannot with truth, speak positively, of an excess of acid being present, unless you see uric acid crystals; and *it is only when free acid is present, in the urine, that alkaline remedies are absolutely necessary.*"

* A fine specimen of Didactic style.

Instead of giving alkaline remedies for the appearance of urate of ammonia in the urine, it would be more rational to consider the cause of this deposit, excess of nitrogenized matter in the blood, and remove it. If it be from indolence and excess of animal food, enjoin exercise and temperance; if from dyspepsia, apply the proper remedy; if from feveror in flammation, in which the nitrogenized tissues are being absorded, and carried out of the system by the kidneys, cure the fever.

*Pus.*—Purulent matter, besides being distinguished from urate of ammonia, by insolubility, by heating the urine in which it is found, is still further identified by allowing it to subside, decanting the supernatent urine and heating it when some degree of coagulation will ensue. The sediment, the supposed pus itself, is to be treated with a solution of caustic potasa, which will dissolve it into a transparent *ropy* fluid.

*Phosphates.*—The earthy phosphates, when present as a sediment, are insoluble by heat, but are soluble in ascetic or nitric acid.

*Blood Globules.*—These, when fresh, are distinguished by tneir red color, and by their form, as seen under the microscope.—Sometimes it is necessary to allow them to settle into a stratum before their character can be well determined. Hematuria points either to Bright's disease, or to calculi in the kidney, yet it is obvious that ulceration or accidental lesion of any parts of the urinary passages may lead to a discharge of blood, and the diagnosis must be made up from all the circumstances of the case.

MICROSCOPIC.

*Spermatazoa.*—Their detection requires a microscope with an acromatic lens, and with a focus not more than one-eight of an inch in length.

*Oxalate of Lime.*—Oxalate of lime crystals, indicative of oxaluria, are found mostly in the form of microscopic octohedra* at the bottom of the vessel in which the urine has been sometime standing. These crystals may be similated by octohedral or cubical crystals of muriate of soda. But the distinction is readily made by transferring the crystals to water, where the muriate of soda is immediately dissolved, while the crystals of oxalate of lime are quite insoluble.

5*th. Heat.—Albumen.*—It is presumed that albuminous urine is seldom or never so highly charged with the material as to be viscid like the white of an egg, or even the serum of the blood.—Albumen in urine is coagulated either by heat or by nitric acid. To avoid fallacies, the operator should repeat the experiments with each of these agents. The albumen may vary in quantity, from

*An octohedron is a solid of 8 sides, being two four-sided pyramids, united base and base.

that which produces by heat, a mere opalescence to that which causes consolidification approaching the consistence of hard boiled white of egg. Albuminous urine is a characteristic of Bright,s disease of the kidneys, but it may occur in the other diseases, esspecially if it be accompanied by pus.

6*th. Evaporation.—Urea.*—If urine is to be evaporated for the purpose of procuring the urea, the process should be conducted over a water-bath, never allowing it to boil. The tin capsules described in this paper, containing about 1,000 grs. of urine, and placed over the water-bath, of the little furnace, answer well for heating and concentrating urine, but they should never be used where acid re-agents are to be applied. When this is to be done, the material should be first transferred to glass, as into a watch crystal.

The chief object of concentrating the urine, is for the purpose of ascertaining the quantity of urea. It the urine have a high specific gravity, it should be evaporated to about one-half its bulk ; if it be watery, and of low specific gravity, it should be concentrated still further. If on cooling, there be much sediment, it should be filtered through thin muslin. To the clear liquid thus obtained, add, when cold, about one-third of its bulk, of pure nitric acid, which answers best, if it be white, and clear of red fumes, at the same time stirring the mixture with a glass rod, or a splinter of window glass. If possible, the liquid should be made ice cold, after this mixture. The nitrate of urea will crystalize in minute pearly scales, sometimes clustering themselves in granules. I generally conduct this process in a large watch-glass, standing on a piece of ice. After a sufficient time has been allowed for cooling and for perfect crystalization, the superabundant liquid may be decanted, by using a strip of glass to restrain the crystals from running off. After this draining, the crystals may be thrown upon a piece of filtering or blotting paper, laid on window glass, cooled by ice underneath, and be again more completely drained, by inclining the plate. It should be finally dried, by enfolding it in blotting paper, and pressing it closely. All of these operations should be concluded rapidly, with strict attention to keeping the materials ice cold, otherwise the crystals of nitrate of urea will vanish by solution.* It is best not to evaporate the urine more than from one-half to one-third the bulk, or the nitrate will be highly colored. In order to obtain this nitrate as white as possible, it should be washed by a small quantity of *ice cold* water, after draining, and before enfolding in the absorbent paper.

---

*Cold well wat r may be substituted.

[*To be continued.*]

